# A pump-free microfluidic device for integrated multi-functional testing of tumor spheroids

**DOI:** 10.1063/5.0329306

**Published:** 2026-06-22

**Authors:** Eliana Steinberg, Gabriel D. Azulay, Alina Ruiz de Villa, Ouri Schwob, Ofra Benny

**Affiliations:** 1 The Institute for Drug Research, The School of Pharmacy, Faculty of Medicine, The Hebrew University of Jerusalem, Jerusalem, Israel; 2 Pre-Cure Ltd., Jerusalem Bio Park (JBP), Minrav Bldg, Hadassah Ein Kerem Campus, Jerusalem, Israel

## Abstract

Three-dimensional (3D) multicellular *ex vivo* cultures have become central tools for cancer research and drug testing under physiologically relevant conditions. Organ-on-a-chip technologies based on microfluidics provide platforms for culturing and analyzing 3D tissues under flow. However, maintaining long-term continuous perfusion typically requires pumps and complex tubing networks, increasing operational complexity, cost, and limiting scalability for routine use. Pumpless approaches have been explored but often suffer from short flow duration, inconsistent unidirectional perfusion, and frequent reservoir replenishment. Here, we present a vertical pump-free fluidic platform designed for spheroid formation, culture, and biological testing. The system integrates molds for spheroid assembly with a modular 3D-printed culture chamber that allows direct sample access. A stackable cartridge-like design enables parallel assays under identical conditions, while the incorporation of commercially available syringes as structural elements improves standardization and reduces the footprint. Continuous perfusion over several days is achieved using a hydrogel-based flow resistor that generates passive pressure gradients. Using three human ovarian cancer cell lines (Ovcar-3, A2780, and Ovcar-8), we demonstrate the formation of uniform spheroids that maintain viability and metabolic activity for up to one week within the 3D-printed cartridges. Drug response was evaluated using paclitaxel, with measurable effects on spheroid growth and invasion. Flow simulations and experimental measurements confirm stable perfusion for approximately 3 days, followed by a gradual decline until cessation at day 7. Overall, this pump-free platform provides a scalable, modular solution for controlled 3D culture and multi-functional assays without external pumping systems.

## INTRODUCTION

Three-dimensional (3D) multicellular *ex vivo* cultures have become valuable models in cancer research and therapeutic screening because they better recapitulate key aspects of the *in vivo* tumor microenvironment, such as cell–cell and cell–matrix interactions, oxygen and nutrient gradients, and intrinsic treatment resistance, compared to conventional monolayer cultures.^1–3^ As a result, 3D models enable more physiologically relevant drug responses and are increasingly used for preclinical testing. In the context of diagnostics, multicellular spheroids serve as a compelling model, providing a minimal yet physiologically tissue-like system.^4,5^ Introducing flow to 3D cultures further improves their relevance by better reflecting *in vivo* transport and microenvironmental conditions.^6–9^ Microfluidic organ-on-a-chip platforms are therefore well suited for these applications, offering perfusion with controlled delivery of test compounds and supporting 3D culture assembly, long-term maintenance, and downstream analysis. Precise control of shear forces and nutrient-waste exchange also makes these systems attractive for parallelization and automation.^10,11^ Despite these advances, achieving the scalability required for high-content screening in research or industry remains a major barrier to adoption. Most platforms rely on external pumps (syringe, peristaltic, or pressure-driven) and extensive tubing to drive flow. Conventional pump-based systems require bulky peripheral equipment and a continuous power supply. Multiple components, including tubing and connectors, are required to interface pumps with the device, increasing the risk of air-bubble formation and introducing potential leakage points that can compromise culture stability. This increases system complexity and cost and limits portability and parallel operation.^12–16^

Consequently, conventional pump-based microfluidic systems are rarely used for routine biological assays or industrial-scale applications, emphasizing the need for self-contained, compact, pump-free solutions.

Although several pump-free approaches have been reported, including gravity-driven and capillary-driven platforms, they often provide limited flow stability and low throughput. Capillary-driven platforms are typically restricted to short-duration assays of a few minutes, whereas gravity-driven systems can support longer operation but require frequent reservoir replenishment during extended operation, often every 16–48 h.^17–24^ Pump-free systems most commonly used for cell culture are rocker-based platforms that rely on recirculating medium. However, these systems generate bidirectional perfusion that is less physiologically relevant than unidirectional flow.^25–32^

In our previously published work,^33^ we demonstrated the integration of spheroid culture within a 3D-printed microfluidic chip while preserving direct sample accessibility. Although 3D-printing provides a rapid, flexible, and low-cost alternative to photolithography and enables complex 3D microfluidic geometries, including along the vertical axis,^34–36^ dependence on external pumps and associated tubing still poses a key limitation to scalability.

In this regard, the platform presented here is not intended to replace conventional microfluidic platforms, particularly in applications requiring highly controlled flow profiles, but rather to provide a simple, pump-free alternative for low-flow perfusion and assays. Here, we combine 3D-printing with a new modular design and introduce a customized, vertical pump-free flow system tailored for spheroid culture and multifunctional assays. The platform uses agarose microwells housed within a template to generate homogeneous spheroids inside a 3D-printed culture chamber. This chamber is built for convenient sample accessibility and uses a stackable cartridge-based architecture that enables multiple assays under identical culture conditions. The chamber was designed in a vertical format to integrate standard commercially available 10 ml syringes as structural components, making the platform compact, simple to operate, cost effective, and broadly accessible. To generate passive perfusion, the chamber outlet is connected to a 10 ml syringe filled with hydrogel, which functions as a flow resistor to slow the flow and maintain stable long-term perfusion without external pumps. The modular chamber's biocompatibility and suitability for simultaneous multiple assays were validated using three human ovarian carcinoma cell lines, Ovcar-3, A2780, and Ovcar-8, to assess cell-line-specific variation. Across all lines, homogeneous spheroids formed reliably and maintained viability and metabolic activity over 7 days within the 3D-printed cartridges. Furthermore, spheroid growth and invasion responses to paclitaxel were monitored over one week, showing treatment-dependent inhibition of both spheroid expansion and invasive behavior. Metabolic activity and spheroid growth of Ovcar-8 spheroids were compared under flow vs static conditions, with no statistically significant differences observed. Finally, flow velocity simulations together with volumetric flow measurements confirmed a continuous low-velocity flow profile, consistent with interstitial-like transport conditions. Stable flow was maintained for 3 days, followed by a gradual decrease over the subsequent 3 days, with flow ceasing on day 7, confirming multi-day perfusion capability.

Taken together, these results demonstrate that our compact, pump-free prototype system addresses key limitations of organ-on-a-chip platforms by eliminating the need for external pumps and tubing, preserving direct sample access, and enabling the integration of multiple functional assays within a single device.

## RESULTS

### Development of a spheroid culture chamber for convenient sample accessibility

**FIG. 1.**
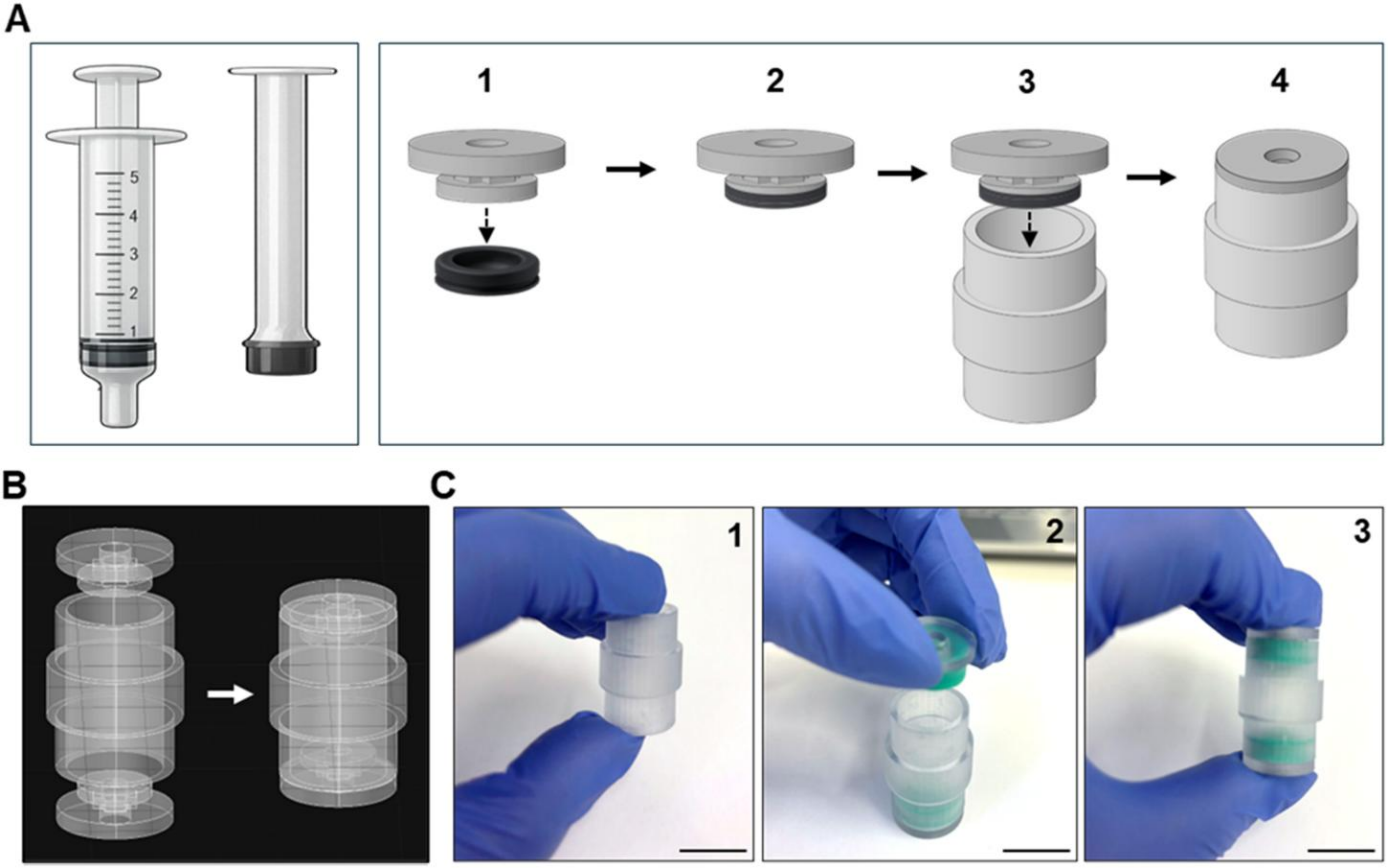
Spheroid culture chamber with convenient sample accessibility. (a) Left: schematic of a syringe sealed by its plunger; the same principle is applied to the chamber. Right: AutoCAD^®^ “conceptual” view of the sealing process. (1) and (2) A rubber gasket from a 10-ml syringe plunger is mounted onto a 3D-printed plunger. (3) and (4) The printed plunger is inserted to seal the 3D-printed culture chamber, enabling convenient opening/closing and sample access. (b) AutoCAD^®^ “x-ray” view showing the chamber sealed from both the top and bottom. (c) Sealing of a 3D-printed culture chamber using custom-designed plungers as depicted in the representative images. (1) Open culture chamber prior to sealing. (2) Chamber sealed at the top and bottom with 3D-printed plungers to ensure a tight closure. (3) Fully sealed culture chamber (scale bar = 19 mm).

To enable spheroid growth and ensure convenient accessibility to samples when needed, a 3D-printed culture chamber was developed [Fig. 1(a)]. The design concept was inspired by the mechanism of a standard syringe and plunger. A 3D-printed plunger was fabricated with the same dimensions as a conventional syringe plunger, allowing a rubber gasket from a 10 ml syringe to be fitted onto it. This configuration enables effective sealing of the 3D-printed culture chamber while allowing convenient opening and closing for sample access, preventing leakage, and enabling easy removal and replacement of spheroids for imaging and further analysis. Figures 1(b) and 1(c) present an internal (“x-ray”) view and macroscopic images of the assembled culture chamber sealed from both the top and bottom, enabling subsequent connection to the remaining components of the pumpless device.

### Template design provides an efficient platform for generating spheroids and assessing viability

**FIG. 2.**
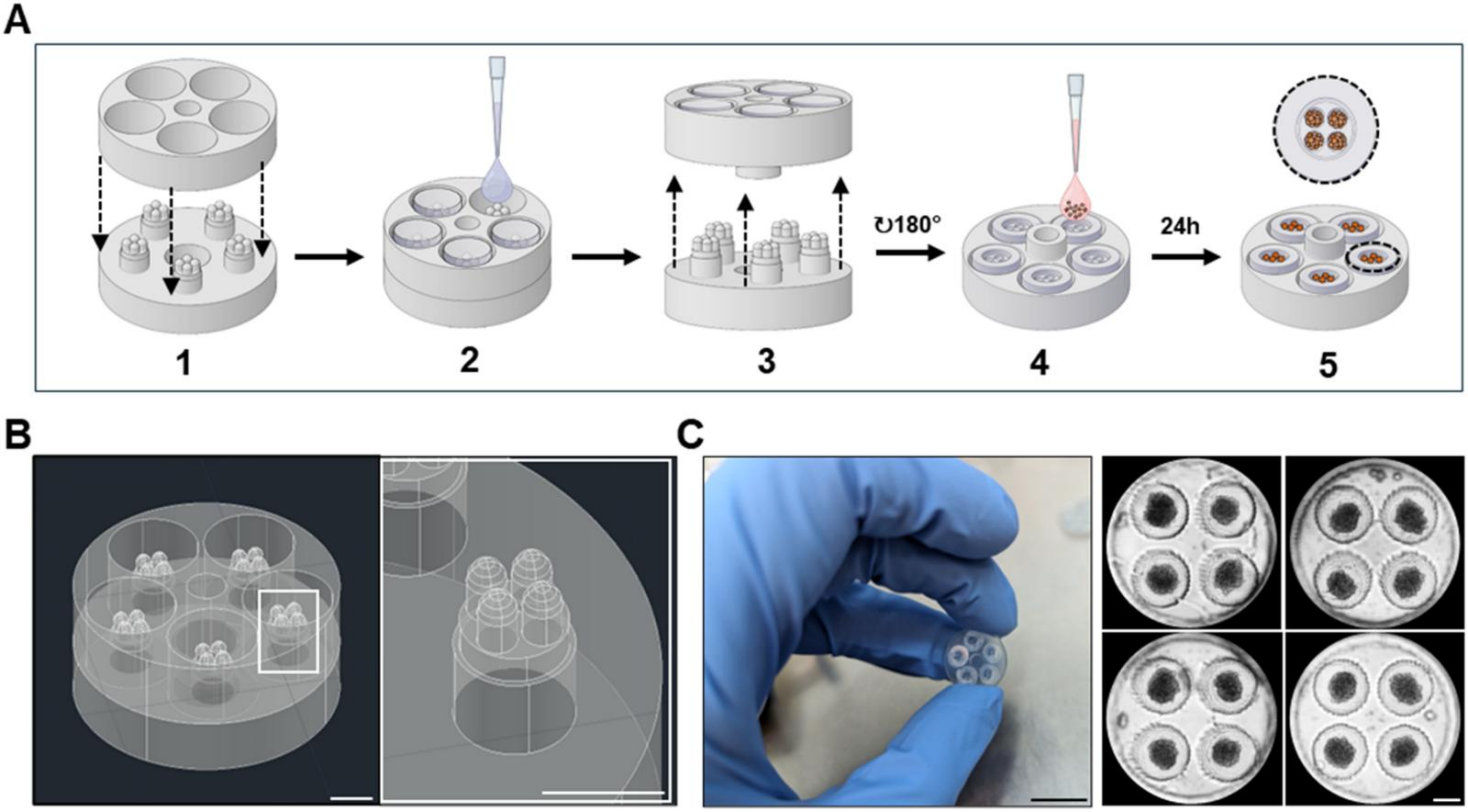
Template design provides an efficient platform for generating spheroids and assessing spheroid viability. (a) AutoCAD^®^ design of the 3D-printed template generating five individual molds, each comprising four microwells designed for spheroid formation using the ultra-low-attachment technique. (1) The top part of the template is placed onto the bottom template. (2) UltraPure™ agarose is pipetted into the template. (3) and (4) The top part is disconnected and turned over, and cells are pipetted into the molds. (5) Spheroids are formed after 24 h of seeding. (b) AutoCAD “x-ray” view showing the template used to form molds for spheroid seeding (scale bar = 2.5 mm). (c) On the left, representative images of agarose molds formed in the 3D-printed template (scale bar = 14.5 mm). On the right, Ovcar-8 spheroids formed in the generated agarose molds, 24 h after seeding 5000 cells/microwell (scale bar = 0.4 mm; n = 12).

Handling and transferring multicellular spheroids remains labor-intensive and technically challenging, particularly when integrating them into microfluidic devices.^10^ To simplify spheroid formation and facilitate subsequent transfer into the flow device, a complementary 3D-printed template was developed. The design generates five individual molds, each containing four microwells for the formation of four spheroids, yielding a total of 20 spheroids per template, using the ultra-low-attachment technique^37^ [Fig. 2(a)]. Prior to cell culture, all 3D-printed parts and commercial components were sterilized by ethanol rinsing followed by UV exposure. The upper and lower templates were aligned, after which UltraPure™ agarose was pipetted into the wells. After solidification, the top part was gently removed, inverted, and seeded with cells in a droplet of culture medium. Following 24 h of incubation, spheroids were formed, and the entire template containing the spheroids could be transferred directly into the flow device without additional manipulation, as it was specifically designed to fit the device configuration. A magnified “x-ray” view of the microwell structure in which spheroids are formed is shown in Fig. 2(b), while Fig. 2(c) presents representative agarose molds and Ovcar-8 homogeneous spheroids formed after 24 h.

### Spheroid viability and metabolic function assessed using the designed template

**FIG. 3.**
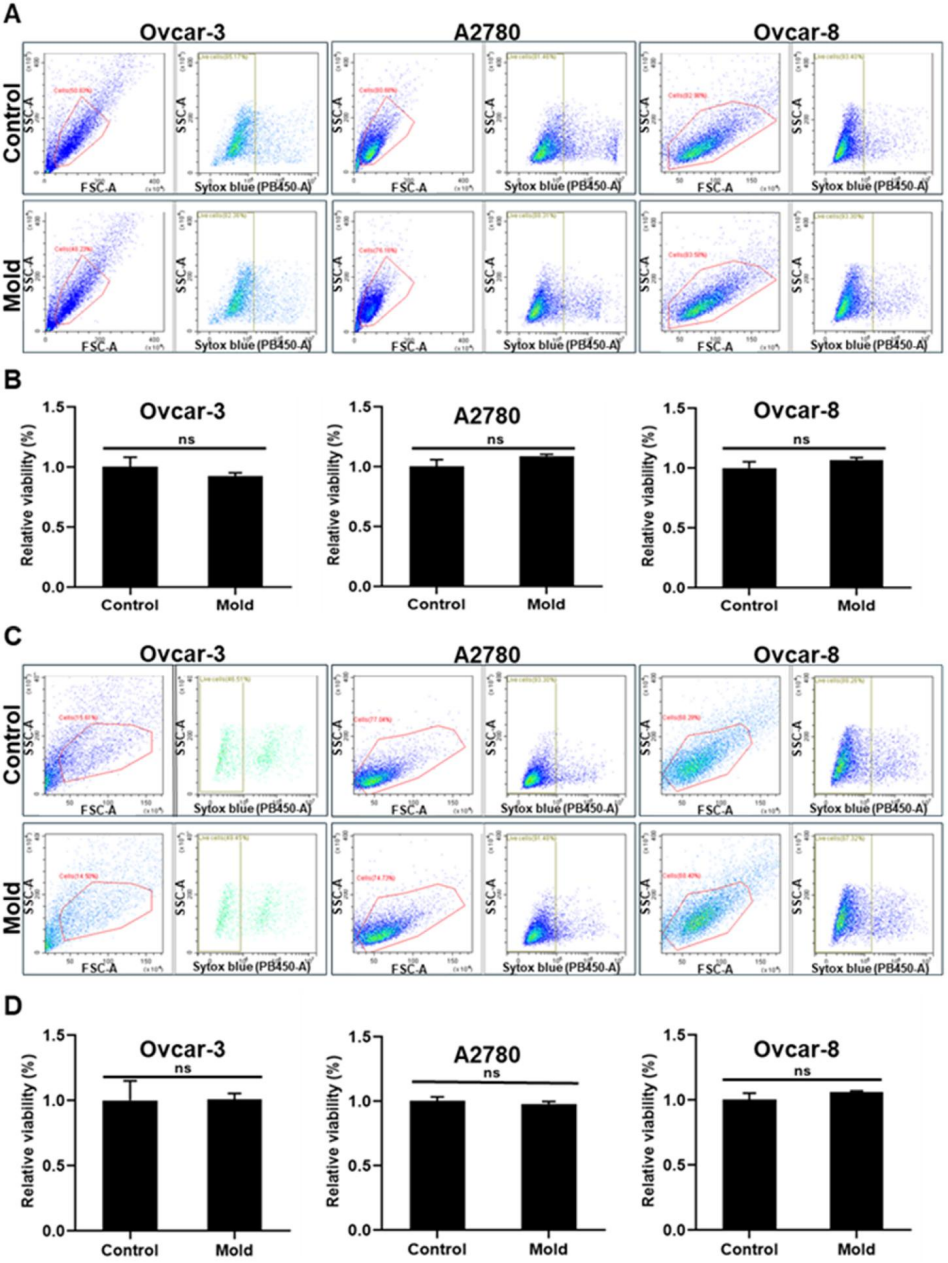
Assessment of spheroid viability and metabolic function using the template design. (a) Representative flow cytometry plots acquired on a CytoFLEX flow cytometer showing the gating strategy and viability assessment of ovarian cancer spheroids (Ovcar-3, A2780, and Ovcar-8) 24 h after culturing in control or mold conditions. On the left, side scatter (SSC-A) and forward scatter (FSC-A) plots illustrate all recorded events and the gating used to identify spheroid populations. On the right, SYTOX^®^ Blue fluorescence (PB450-A, x-axis) vs side scatter (SSC-A, y-axis) indicates live (SYTOX-negative) and dead (SYTOX-positive) cells (n = 4). (b) Metabolic activity of Ovcar-3, A2780, and Ovcar-8 spheroids was evaluated 24 h after placement in control and mold conditions using a WST-1 cell viability assay. Absorbance was measured at 450 nm using a Wallac 1420 VICTOR plate reader. Data represent the relative metabolic activity of spheroids under the indicated conditions compared with control (n = 6–8). (c) Representative flow cytometry plots acquired on a CytoFLEX flow cytometer showing the gating strategy and viability assessment of ovarian cancer spheroids (Ovcar-3, A2780, and Ovcar-8) 7D after culturing in control or mold conditions. On the left, side scatter (SSC-A) and forward scatter (FSC-A) plots illustrate all recorded events and the gating used to identify spheroid populations. On the right, SYTOX^®^ Blue fluorescence (PB450-A, x-axis) vs side scatter (SSC-A, y-axis) indicates live (SYTOX-negative) and dead (SYTOX-positive) cells (n = 4). (d) Metabolic activity of Ovcar-3, A2780, and Ovcar-8 spheroids was evaluated 7D after placement in control and mold conditions using a WST-1 cell viability assay. Absorbance was measured at 450 nm using a Wallac 1420 VICTOR plate reader. Data represent the relative metabolic activity of spheroids under the indicated conditions compared with control (ns = not significant; n = 6–8). Results are presented as mean ± SEM.

To evaluate the biocompatibility and functionality of the newly designed template for metabolic testing, Ovcar-3, A2780, and Ovcar-8 spheroids were seeded in agarose molds under two static conditions: control, in a standard 96-well plate, and in the mold, within the 3D-printed template. Cell viability and metabolic activity were assessed at 24 h and 7 days after seeding. Viability was evaluated by flow cytometry following SYTOX^®^ Blue staining, which labels non-viable cells [Figs. 3(a) and 3(c)]. After 24 h, viabilities in control and mold conditions were comparable: Ovcar-3 (85% vs 82%), A2780 (81% vs 88%), and Ovcar-8 (93% vs 93%), with no statistically significant differences. After 7 days, similar results were observed: Ovcar-3 (46% vs 49%), A2780 (93% vs 91%), and Ovcar-8 (88% vs 87%), again with no significant differences between the two conditions.

Metabolic activity, determined using the WST-1 assay, confirmed these findings, showing no significant difference between control and mold conditions at either 24 h or 7 days [Figs. 3(b) and 3(d)].

### Mold design enables efficient generation of spheroids and evaluation of their size and invasion capacity

**FIG. 4.**
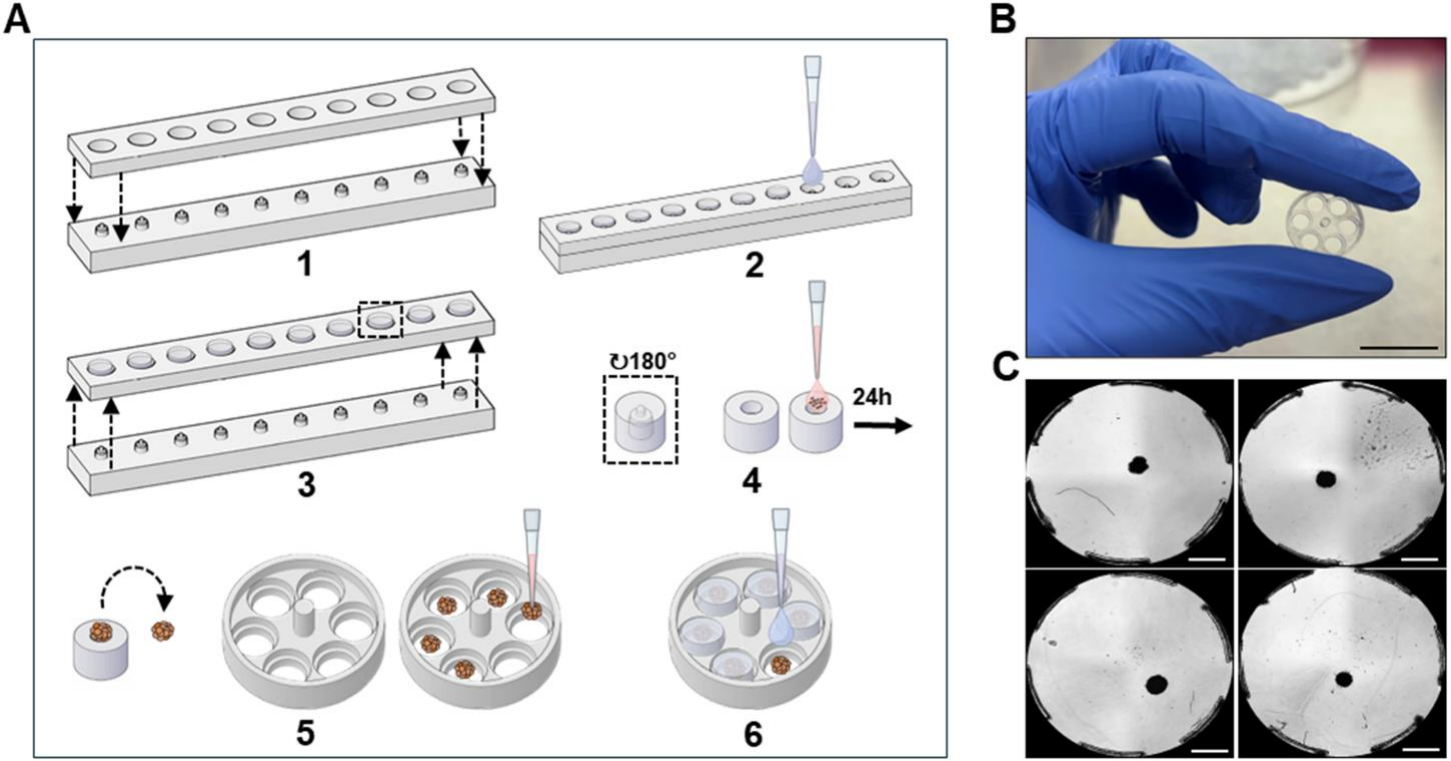
Mold design enables efficient generation of spheroids and evaluation of their invasion capacity. (a) AutoCAD^®^ design and workflow for generating one-microwell templates for spheroid formation and subsequent transfer into a specifically designed 3D-printed invasion mold. (1) The top part of the template is aligned and placed onto the bottom template. (2) Ultrapure agarose is pipetted into the assembled template to create the microwell molds. (3) and (4) The top part is detached; the molds are removed, inverted, and used for spheroid seeding by pipetting cell suspensions into the wells. (5) Compact spheroids form within 24 h and are subsequently transferred into a 3D-printed mold for invasion analysis. (6) Matrigel**^®^** is applied over the spheroids, providing a 3D ECM to support and enable invasion. (b) Representative image of the invasion 3D-printed mold (scale bar = 1.5 cm). (c) Ovcar-8 spheroids formed in agarose microwell molds were transferred to the invasion mold 24 h after seeding 5000 cells per microwell (scale bar = 1 mm; n = 12).

To test multiple assays in parallel under identical conditions, we employed two functional assays, 3D invasion and growth, which are widely used to assess tumor aggressiveness and drug responsiveness.^38^ To integrate these assays into our flow device, we designed a one-microwell mold template that enables the formation of a single spheroid per well, yielding a total of 10 spheroids per template. The mold consists of top and bottom parts; once assembled, UltraPure™ agarose was pipetted into the wells to form the microwell structures [Fig. 4(a)]. Following solidification, Ovcar-8 cells were seeded at a density of 5000 cells per microwell, leading to the formation of uniform spheroids within 24 h. The resulting spheroids were subsequently embedded in Matrigel^®^, which served as a 3D extracellular matrix (ECM) to support invasion. Imaging within the invasion molds enabled visualization and quantitative evaluation of invasive behavior [Figs. 4(b) and 4(c)].

### Evaluation of spheroid size and invasion capacity using the designed mold

Paclitaxel, a standard first-line chemotherapeutic agent in ovarian cancer, commonly used with platinum-based therapy,^39^ was selected for proof-of-concept testing in our model. To evaluate the applicability of our designed mold for monitoring spheroid growth and invasion, Ovcar-8 cells were seeded into the microwells at a density of 5000 cells per microwell. After 24 h, the formed spheroids were transferred to the invasion mold, overlaid with Matrigel^®^, and cultured under three static conditions: untreated control, immediate paclitaxel treatment (1 *μ*M added at transfer), and delayed paclitaxel treatment (1 *μ*M added 2 days after embedding). The delayed-treatment condition was included to test whether ECM interaction and the onset of invasion alter paclitaxel efficacy by comparing treatment initiated at the time of embedding with treatment initiated after cells had initiated invasion, a transition known to activate survival programs and phenotype shifts that can reduce chemotherapy sensitivity.^40,41^ The selected paclitaxel concentration was based on IC_50_ assays performed on Ovcar-8 spheroids treated with increasing paclitaxel concentrations [Fig. S1(a)].

**FIG. 5.**
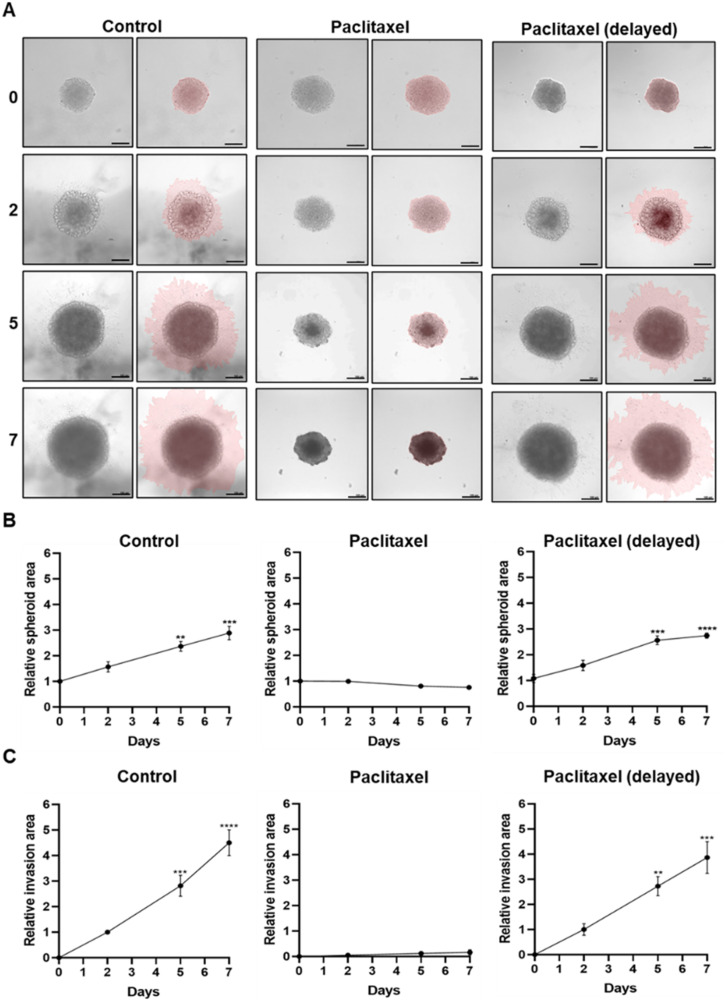
Evaluation of spheroid size and invasion capacity using the designed mold. (a) Representative brightfield images of Ovcar-8 spheroids embedded in Matrigel 24 h after seeding and imaged using a Nikon Eclipse Ti microscope immediately after transfer and after 2, 5, and 7 days. Spheroids were cultured under three conditions: control (no treatment), paclitaxel (1 *μ*M) added immediately after transfer, and paclitaxel (delayed), in which treatment was added 2 days after embedding. Left: brightfield images; right: whole-spheroid outlines generated using NIS-Elements software (scale bar = 200 *μ*m). (b) Quantification of spheroid area over time measured using NIS-Elements immediately after embedding and after 2, 5, and 7 days. Data represent relative spheroid area under the indicated conditions, normalized to the size measured immediately after transfer (time 0). (c) Quantification of spheroid invasion area determined by measuring the total area (including invading cells) and subtracting the spheroid core area using NIS-Elements. Data represent relative invasion area under the indicated conditions, normalized to the area measured immediately after transfer (time 0) (^**^p < 0.01, ^***^p < 0.001, ^****^p < 0.0001; n = 6). Results are presented as mean ± SEM.

Brightfield images were captured immediately after transfer and subsequently at days 2, 5, and 7. Spheroid size and invasion area were quantified using Nikon Imaging Software (NIS)-Elements software [Fig. 5(a)]. Quantification of spheroid area over time is shown in Figs. 5(b) and S1(c), corresponding to the marking scheme in Fig. S1(b). Control spheroids exhibited progressive growth, with average area increases of approximately 57%, 136%, and 188% on days 2, 5, and 7 after ECM embedding, respectively. In contrast, spheroids treated immediately with paclitaxel displayed reduced growth, with their area slightly decreasing by day 2 and decreasing further by approximately 20% and 25% on days 5 and 7, respectively. Spheroids exposed to delayed paclitaxel treatment showed area increases of approximately 58%, 156%, and 174% on days 2, 5, and 7, respectively, closely resembling the growth profile of control spheroids. Invasion capacity was measured by quantifying the total spheroid area, including invading cells, and subtracting the spheroid core area [Figs. 5(c) and S1(d)]. Both control and delayed paclitaxel-treated spheroids exhibited comparable increases in invasion area, with more pronounced expansion observed by day 7 (approximately 145% and 138% increases, respectively). In contrast, spheroids treated immediately with paclitaxel showed minimal invasion throughout the experiment, indicating strong inhibition of invasive behavior under continuous drug exposure.

### Development of a pump-free vertical-flow device for spheroid culture

**FIG. 6.**
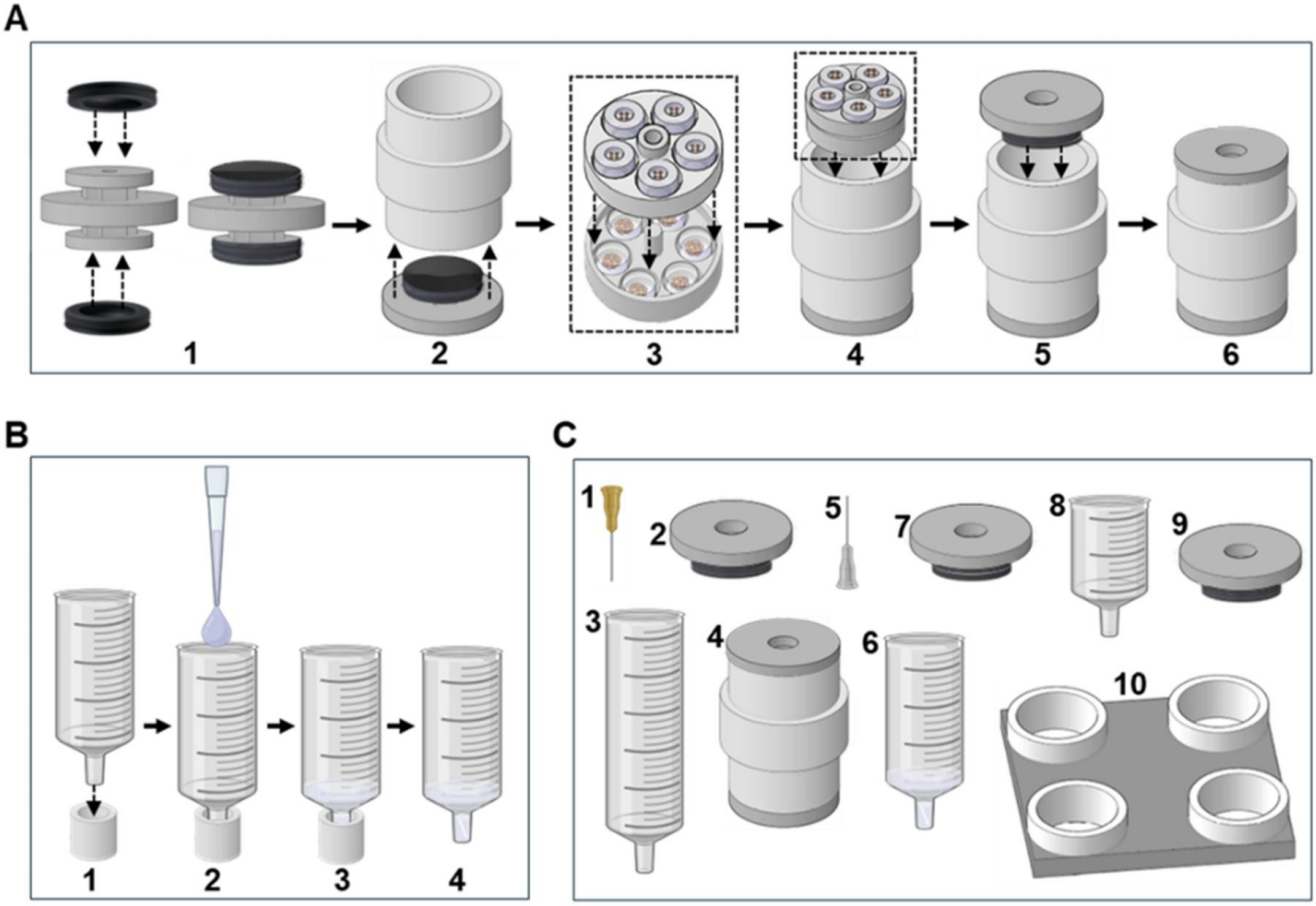

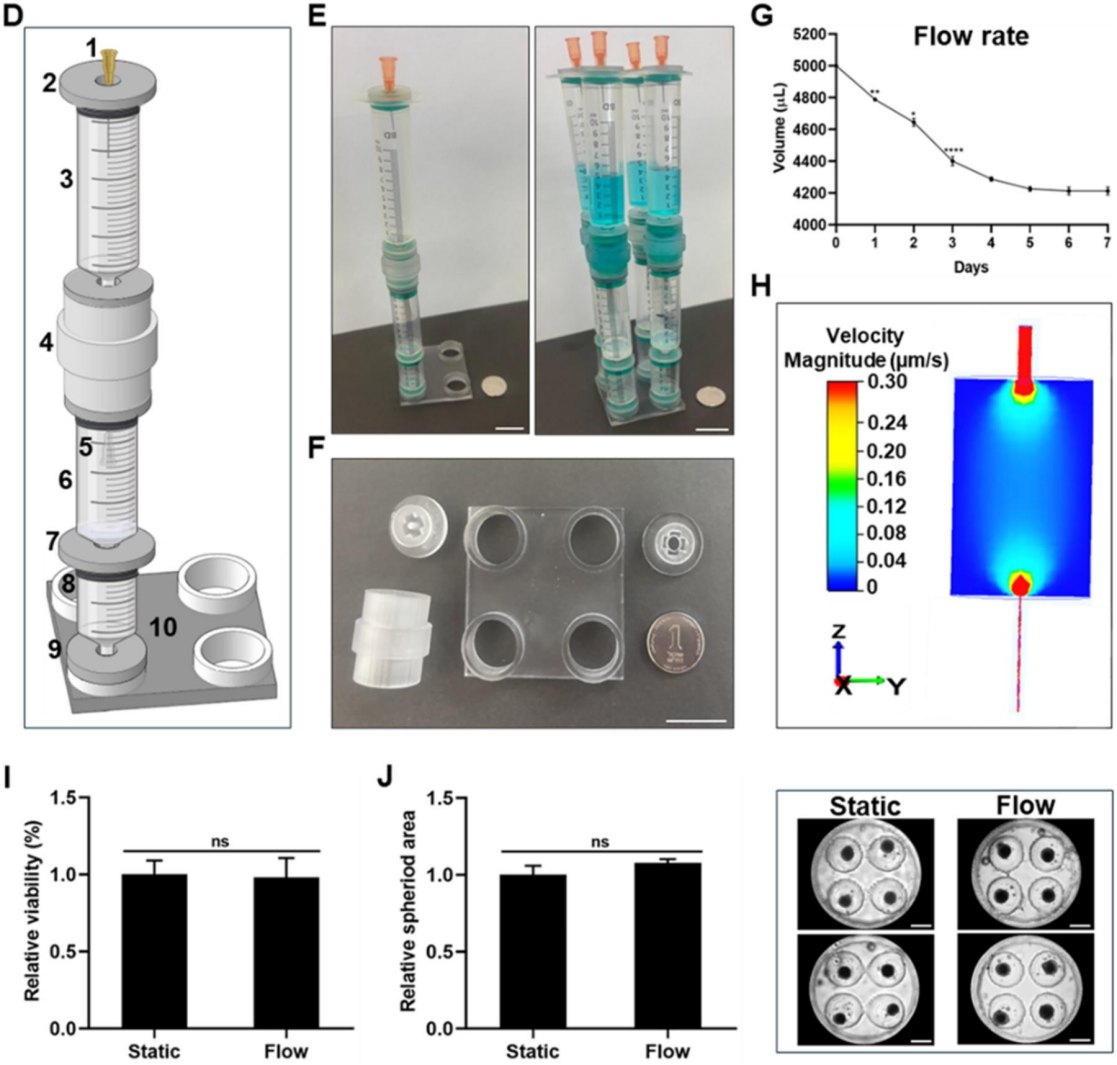
Pump-free vertical-flow device for spheroid culture. (a) AutoCAD^®^ design and workflow for assembling the 3D-printed spheroid culture chamber for simultaneous testing of different spheroid functions. (1) Rubber gaskets from 10 ml syringes are placed on a 3D-printed, custom-designed two-way plunger. (2) The plunger is used to seal the bottom of the culture chamber. (3) Functional testing templates are designed to fit precisely on top of one another. (4) Templates are inserted into the culture chamber. (5) and (6) The chamber is sealed with a custom-designed plunger fitted with a rubber gasket. (b) Workflow for preparing a hydrogel flow barrier serving as a flow resistor. (1) A syringe is placed in a 3D-printed sealing tip. (2) and (3) UltraPure™ agarose is pipetted into the syringe and allowed to solidify. (4) The seal tip is removed, and the syringe is ready for use in the pumpless flow device. (c) and (d) Illustrations showing all components used to assemble the pumpless flow device and the fully assembled device, respectively. (1) Needle inserted into the top custom-designed plunger for air circulation. (2) and (3) Custom-designed plunger used to seal the reservoir syringe. (4) Culture chamber containing templates for functional spheroid testing. (5) Needle at the culture chamber outlet through which media flow. (6) Syringe containing the hydrogel barrier serving as a flow resistor. (7) and (8) Custom-designed plunger used to connect the flow resistor to the waste-collection syringe. (9) and (10) Custom-designed plunger used to attach the pumpless device to a 3D-printed stand supporting up to four devices. (e) Photographs of assembled pumpless devices. Left: an example of a single assembled system; right: four assembled devices filled with de-ionized water (DW) stained with blue food coloring (scale bar = 2 cm). (f) Representative images of the 3D-printed components used to assemble the pumpless device (scale bar = 2 cm). (g) A total of 5 ml of de-ionized water (DW) stained with blue food coloring was added to the reservoir syringes, and pumpless devices were operated for 7 days. Reservoir volume was recorded daily using syringe graduation markings. Data represent reservoir volume over time, with each time point compared to the previous day (n = 4–9). Results are presented as mean ± SEM. (h) Side-view CFD simulation showing velocity magnitude distribution within the culture chamber of the device. (i) Metabolic activity of Ovcar-8 spheroids was evaluated 3 days after placement under static control conditions or in the pump-free flow device using a PrestoBlue™ cell viability assay. Fluorescence was measured at Excitation 530/25 nm and Emission 590/20 nm using a Wallac 1420 VICTOR plate reader. Data represent relative metabolic activity under the indicated conditions, normalized to the static control (n = 4). (j) Quantification of spheroid area after 3 days under static and flow conditions was performed using the NIS-Elements software. Data represent relative spheroid area normalized to static control conditions (n = 4). Representative brightfield images of Ovcar-8 spheroids after 3 days of culture under both conditions are shown on the right and were acquired using a Nikon Eclipse Ti microscope (scale bar = 0.4 mm). Results are presented as mean ± SEM (ns = not significant).

To eliminate the need for external pumps, a gravity-driven vertical-flow device was developed to enable pumpless perfusion for spheroid culture. The workflow for assembling the 3D-printed culture chamber is illustrated in Fig. 6(a). The chamber was designed to accommodate simultaneous testing of multiple spheroid functions under controlled conditions. Two rubber gaskets were installed onto a custom-designed, double-sided 3D-printed plunger matching the dimensions of a standard 10 ml syringe plunger. This plunger served to seal the bottom of the culture chamber, while its double-sided design enabled direct connection of the chamber to other system components in a vertical configuration, minimizing the overall space requirements. Within the culture chamber, two functional testing templates were inserted, each designed to fit precisely in a stacked configuration.

To generate counterpressure and slow the flow, a hydrogel-based flow resistor was constructed. It consisted of a 10 ml syringe filled with 1 ml of molten agarose, which was allowed to solidify at the syringe tip to form a hydrogel-based flow barrier. The syringe was subsequently connected to the bottom of the culture chamber containing the spheroid templates [Fig. 6(b)]. All components required for assembly of the pumpless flow chip are shown in Fig. 6(c), and a schematic of the complete device is presented in Fig. 6(d). The assembled system integrates a combination of 3D-printed and commercial components designed to enable controlled medium circulation without external pumps. The upper plunger functions as an air vent to balance internal pressure, while the culture chamber houses the stacked templates for spheroid assays and connects downstream to the hydrogel-based flow resistor. The agarose at the bottom of the syringe provides stable resistance, ensuring continuous medium exchange through passive pressure differentials. The outlet connects to a waste-collection syringe through additional custom plungers, ensuring unidirectional flow throughout the system. The entire system is assembled in a vertical orientation on a 3D-printed stand, secured via a custom plunger and gasket. Representative images of single and multi-unit setups demonstrate the modular design and reproducibility of the assembly [Fig. 6(e)], while the corresponding 3D-printed components are shown in Fig. 6(f).

Flow rates were quantified by tracking changes in reservoir volume over time using syringe graduation markings. The measured flow rate of the device without reservoir replenishment is presented in Fig. 6(g) and demonstrates a stable flow of ∼200 *μ*l per day (0.139 *μ*l/min) during the first 3 days of operation, followed by a gradual decrease over the following 4 days and complete cessation on day 7. Time-lapse video of the flow rate measurement and representative images of reservoir volume reduction are shown in Figs. S2 and S3(a), respectively.

To assess interstitial-like transport within the device, computational fluid dynamics (CFD) simulations were performed. The CFD results indicated a decrease in flow velocity from the inlet to the culture chamber, with increased velocity at the narrow outlet. The mean velocity within the culture chamber was estimated to be ∼0.01 *μ*m/s [Fig. 6(h)], while absolute pressures remained near atmospheric, with a hydrostatic pressure increase of ∼2.3 mm Hg from the top to the bottom of the culture chamber [Fig. S3(b)]. Together, these results indicate that the device operates under a low-velocity, laminar perfusion regime.

To evaluate the biocompatibility of the fully assembled flow device and compare metabolic activity and spheroid growth under static and perfused conditions, Ovcar-8 spheroids were seeded in agarose molds contained within 3D-printed templates and, after 24 h, cultured either under static conditions in a standard 24-well plate or within the flow device. Cell viability and metabolic activity were assessed after 3 days of culture under these conditions using a PrestoBlue™ cell viability assay [Fig. 6(i)]. Viability under static and flow conditions was comparable, with no statistically significant differences observed. Brightfield images were acquired after 3 days of culture under both conditions, and spheroid size was quantified using the NIS-Elements software [Fig. 6(j)], showing no statistically significant difference between static and flow conditions.

## DISCUSSION

Microfluidic devices designed for culturing biological samples typically rely on pumps to regulate pressure and maintain steady flow. In conventional setups, device assembly involves alignment and bonding of multiple layers, followed by coupling of inlets and outlets to a pump system, usually syringe or peristaltic, to achieve controlled medium flow.^42–44^ While providing precise flow regulation, pump systems substantially increase system complexity, bulkiness, and cost, thereby limiting scalability.^45–47^ Multiple parts and connectors can cause frequent leakage and air bubbles, which disrupt flow and compromise experiments.^15,16^ In many biological assays, such complexity is not strictly required; thus, alternative flow systems have been explored.

Several groups have attempted to reduce reliance on external pumps by developing gravity-driven, rocker-based, or capillary-driven microfluidic systems.^17–32^ For example, Komeya *et al.* utilized hydrostatic pressure and a resistance circuit to achieve flow in a pump-free device for inducing mouse spermatogenesis. However, because the device layers are irreversibly bonded, access to the cultured tissue is limited, and the long built-in resistance circuit increases device size and requires complete refabrication to modify the flow rate.^20^ Tian *et al.* demonstrated a gravity-driven back-and-forth flow system for spheroid culture actuated by a rocker platform.^28^ This strategy can sustain flow over extended periods and is used commercially through designated plates and equipment.^48–50^ However, the resulting oscillatory perfusion is less physiologically relevant than unidirectional flow, which may influence assay outcomes. Other approaches for pump-free flow include capillary-driven devices, mainly used for point-of-care immunoassays. While effective for initiating slow flow, this mode is usually sustained only for minutes.^22,24,51–53^

The pump-free flow platform presented here (Fig. 6) was intentionally designed to be simple, provide easy sample access, and ensure reproducibility. Except for the 3D-printed culture chamber, all components, including syringes, gaskets, needles, and agarose, are commercially available, and assembly does not require specialized equipment. By employing previously validated biocompatible materials for 3D-printing, the system ensures safe and reliable culture conditions for sensitive cell types. This aligns with our earlier work,^33^ where the same resin and fabrication workflow were shown to support long-term cell viability and functional readouts. As 3D-printing is a rapidly developing technology, it is expected to become increasingly accessible and common in many laboratories.^54^ The gravity-based vertical flow configuration and stacked mold architecture further reduce overall dimensions, enabling many chips to be run in parallel, thereby improving scalability. Moreover, the lack of tubing reduces the likelihood of leakage and air bubble formation. This platform is not intended to replace conventional pump-controlled microfluidic systems in applications requiring highly precise flow rates, integrated sensing, or complex multi-channel vascular architectures. Rather, it provides a simple, accessible, and modular alternative for low-flow assays that can benefit from continuous perfusion without the need for complex experimental sequences or device designs. We demonstrate its applicability for spheroid culture and functional testing in settings where ease of use, direct sample access, and parallelization are prioritized.

One of the unique components in our flow system is a hydrogel-based flow resistor that provides stable passive medium perfusion without external pumps. Agarose hydrogel acts as a porous barrier that creates steady resistance, allowing hydrostatic pressure alone to drive slow, continuous flow as the medium passes through the gel.^55,56^ Achieving a flow rate that is slow enough for long-term culture while remaining continuous is a major limitation of pump-free systems. We addressed this by inserting metal needles into the gaskets to reduce channel diameter at the connections and, most importantly, by incorporating a hydrogel barrier as a flow resistor. Without the hydrogel, the flow rate rose to approximately 1 ml per hour, which is too high for long-term cell culture, confirming the critical role of the resistor. The resulting flow remained steady at approximately 200 *μ*l per day during the first 3 days, gradually decreased over the next 3 days, and ceased on day 7. This initial flow rate is comparable to that reported by Limjanthong *et al.*, who used a slow tilting table to generate perfusion for long-term cell culture.^26^ The CFD-predicted mean velocity of ∼0.01 *μ*m/s falls within the lower range of the reported tumor interstitial fluid velocities (typically in the range of ∼0.01 to 10 *μ*m/s).^8^ Under these conditions, the resulting shear stresses acting on the tumor spheroids are expected to be minimal. Such low-velocity, laminar flow is consistent with interstitial-like transport in solid tumors. In contrast, many alternative pump-free devices support only short perfusion durations (minutes to hours) or require frequent reservoir replenishment to sustain longer operation.^17–24^ For example, a droplet-dispensing pump-free device supporting 6 days of culture required refilling every 16 h,^18^ and a hydrostatic-pressure system operated for 12 days only by manually topping up the inlet reservoir every 24 h.^19^ Since manual reservoir replenishment is not ideal, our design reduces the frequency of intervention, with refilling needed only every 3 days to sustain the initial flow rate. Future studies may investigate how varying agarose concentration affects flow rate and medium exchange dynamics, potentially enabling straightforward tuning of the platform for different tissue models.

The modular 3D-printed culture chamber (Fig. 1) provides a key advantage of direct access to the culture region, enabling sample retrieval, imaging, or reagent exchange through easy and quick disassembly and assembly of the device. Beyond accessibility, the ability to extract rich biological information from the same sample is a major advantage, particularly in oncology, where tumor behavior manifests through multiple functional phenotypes.^57,58^ To assess anticancer drug effects, we quantified viability, metabolic activity, growth, and invasion. Because these readouts are especially informative, we integrated them into the device as interchangeable cartridges that stack precisely on top of one another (Figs. 2 and 4). This multiparametric format enables several complementary readouts from the same small sample under identical conditions, which is especially valuable for patient-derived material that is typically scarce and difficult to obtain. Depending on the application, a specific assay mold can be installed in one or both cartridges, enabling flexible, modular experimental design and multiparametric testing. Additional assays, such as angiogenesis or immune-response studies, could be incorporated in future work. Comparable modular approaches have been reported, but most rely on complex fluidic interfaces or external valves and actuators to maintain separate microenvironments, limiting ease of use and scalability.^59–62^

It is well established that introducing flow to cancer models can enhance physiological relevance, as fluid dynamics play a major role in tumor progression.^6–9,63^ In the present work, we assessed metabolic activity and spheroid growth in Ovcar-8 spheroids under both flow and static conditions and found no statistically significant differences [Figs. 6(i) and 6(j)]. Notably, the objective of this study was not to further investigate the biological effects of flow, which would require evaluating a broader set of assays and treatment conditions to determine how responses differ between flow and static environments, but rather to validate the platform's biocompatibility, demonstrate performance comparable to standard static cultures, and highlight its accessibility and capacity to support multiple functional assays within a single modular system. This proof-of-concept validation was performed using spheroids derived from three ovarian cancer cell lines, Ovcar-3, A2780, and Ovcar-8, demonstrating the platform's ability to support a focused set of functional readouts across representative models with cell-line diversity. To ensure the system was not cytotoxic, we compared viability and found that spheroids cultured within the 3D-printed molds maintained values comparable to those grown under standard static conditions (Fig. 3). This indicates that the device is biocompatible and does not adversely affect ovarian cell health or metabolic function over time. These results supported the next step of testing additional cell functions in the system under drug treatment. Paclitaxel treatment experiments (Fig. 5) demonstrated the capacity to monitor temporal changes in spheroid size and invasiveness, which are key parameters for assessing chemotherapeutic efficacy.^41,64,65^ As expected, untreated spheroids increased in size and invaded the surrounding ECM, while spheroids receiving immediate paclitaxel treatment showed minimal change. We included a delayed treatment condition to determine whether initiating paclitaxel after invasion had begun alters treatment response, given that early matrix interaction can alter drug sensitivity.^40,41^ Interestingly, we found that spheroids exposed to delayed paclitaxel treatment were comparable in size and invasiveness to untreated controls. One possible explanation is that by the time paclitaxel was added, the spheroids had already become denser, which could limit drug penetration and reduce treatment effectiveness relative to immediate treatment. Further investigation of this timing-dependent resistance would be of interest in future studies.

In summary, this work presents a pump-free fluidic system designed to support reproducible spheroid formation and culture with convenient sample accessibility, and the potential for quantitative functional testing under controlled flow conditions. By replacing external pumps with a passive hydrogel flow resistor, the platform eliminates the need for complex equipment while maintaining stable medium exchange suitable for cell functional assays such as viability and invasion testing. Constructed primarily from components readily available in any standard laboratory, the platform is also easily applicable and straightforward to implement. The stackable cartridge-based design introduces exceptional modularity, allowing customized configurations for simultaneous multi assay experiments and offering the potential to expand toward organotypic or co-culture models. Despite these advantages, the system currently remains at the level of laboratory operation, and several technical challenges still remain. Occasional partial channel occlusion can occur, leading to flow interruptions and potentially affecting experimental consistency. In a few devices, air leakage altered the pressure resistance, resulting in accelerated drainage. Therefore, future work will focus on improving system robustness and reducing variability to enhance reproducibility and operational reliability. Future developments may also incorporate sensors for real-time metabolic monitoring or gradient generators for microenvironmental control, further broadening the system's applications in cancer biology, drug screening, and tissue engineering.

## METHODS

### 3D designing and printing

Device designs were created using Autodesk AutoCAD^®^ (Autodesk, San Rafael, CA, USA) and exported as stereolithography (STL) files. The final STL designs were imported into Asiga Composer software (Asiga, Sydney, Australia) for printing. All molds and devices were fabricated using an Asiga Max digital light processing (DLP) stereolithography printer (Asiga, Sydney, Australia) equipped with a 385 nm UV LED light source. Printing was performed with FREEPRINT^®^ Ortho resin (Detax GmbH, Baden-Württemberg, Germany). After printing, the parts were detached from the build platform, and uncured resin was removed by rinsing in pure ethanol. Printed components were subsequently UV-cured for 1 h using a LED curing unit (Dreve, Germany). All 3D-printed parts and commercial components were sterilized by immersion in 100% ethanol overnight, followed by UV exposure for 30 min prior to assembly.

### Cell culture

Human ovarian cancer cell lines Ovcar-3, A2780, and Ovcar-8 were kindly provided by Reich's laboratory at the Hebrew University of Jerusalem. Ovcar-3 and Ovcar-8 cells were originally acquired from ATCC (Manassas, VA, USA). A2780 cells were obtained from ECACC (Salisbury, UK). Cells were routinely tested and confirmed to be mycoplasma-free using an EZ-PCR Mycoplasma Test Kit (Biological Industries, Israel) and were used for experiments up to passage 20. Cells were maintained in culture medium supplemented with 10% fetal calf serum (FCS; Gibco, Brazil) and 1% penicillin–streptomycin (P/S) and kept in a humidified incubator at 37 °C with 5% CO_2_. Ovcar-8 and A2780 cells were cultured in Roswell Park Memorial Institute (RPMI-1640) medium (Sigma-Aldrich, USA), while Ovcar-3 cells were maintained in Dulbecco's Modified Eagle Medium (DMEM) (Sigma-Aldrich, USA).

### Spheroid formation using ultra-low attachment microwells

Complementary 3D-printed templates were designed to produce five molds, each containing four microwells, enabling the formation of four spheroids per mold. UltraPure™ agarose hydrogel (4%) (Corning, NY, USA) was used to cast the molds. Following a 3-min incubation at room temperature (RT), the templates were removed, leaving defined microwells for subsequent cell seeding. Ovcar-8 cells were seeded at a density of 5000 cells per microwell. Spheroid formation was monitored and images were acquired 24 h after seeding using a Nikon Eclipse Ti microscope (Tokyo, Japan).

### Viability and metabolic function testing of spheroids in 3D-printed templates

For viability and metabolic function assays, Ovcar-3, A2780, and Ovcar-8 cells were seeded in UltraPure™ agarose molds at a density of 5000 cells per microwell under two conditions: (1) control, where molds were placed within standard 96-well culture plates, and (2) mold, where molds were housed within 3D-printed templates fabricated as described above. After 24 h and 7 days of incubation, spheroid viability and metabolic activity were assessed.

For viability testing and flow cytometry analysis, spheroids were gently pipetted from the agarose molds into standard culture 96-well plates and then dissociated into single cells via trypsinization using trypsin–EDTA (Sigma-Aldrich, MO, USA) with a 5-min incubation, followed by neutralization with culture medium. Cells were stained with SYTOX^®^ Blue Dead Cell Stain (Invitrogen, USA) according to the manufacturer's instructions at a final concentration of 5 *μ*M, and incubated for 5 min at room temperature, protected from light. Fluorescence was measured using a CytoFLEX flow cytometer (Beckman Coulter, USA) with a 450/45 nm detector (violet laser) and a 440/40 nm bandpass filter. Data analysis was performed using the FlowJo software (Tree Star, Ashland, OR, USA).

For metabolic function testing, the Water-Soluble Tetrazolium-1 (WST-1) reagent (Sigma-Aldrich, MO, USA) was applied according to the manufacturer's protocols. Absorbance was measured at 450 nm using a Wallac 1420 VICTOR plate reader (PerkinElmer Life Sciences, Shelton, CT, USA).

### Generation of spheroids for evaluating invasion capacity

Ovcar-8 spheroids were generated as described above, using 3D-printed custom-designed templates containing single-microwell molds for spheroid formation. After 24 h of incubation with 5000 cells per microwell, spheroids were formed and subsequently transferred by pipetting into specifically designed 3D-printed invasion molds. A total of 50 *μ*l of Matrigel^®^ Matrix was added to each well and allowed to polymerize for 30 min in a humidified incubator at 37 °C. The polymerized Matrigel served as a 3D ECM to support and enable spheroid invasion.

### Evaluation of spheroid size and invasion capacity using the designed mold

Ovcar-8 spheroids were formed and embedded in Matrigel^®^ as described above, 24 h after seeding. Spheroids were imaged using a Nikon Eclipse Ti microscope immediately after transfer and after 2, 5, and 7 days. Spheroids were cultured under three conditions: control (no treatment), paclitaxel added immediately after transfer, and paclitaxel (delayed), in which treatment was applied 2 days after embedding. Paclitaxel (Cayman Chemical, Ann Arbor, MI, USA) was dissolved in DMSO (Dimethyl sulfoxide) and used at a final concentration of 1 *μ*M, unless otherwise stated. Quantification of spheroid area over time was performed using NIS-Elements software (Nikon, Japan).

To determine the optimal paclitaxel concentration for the invasion assay, the metabolic activity of Ovcar-8 spheroids (seeded at 5000 cells per microwell in four-microwell molds placed in 96-well culture plates) was evaluated 7 days after paclitaxel addition at increasing concentrations using a WST-1 cell viability assay. Absorbance was measured at 450 nm using a Wallac 1420 VICTOR plate reader.

### Hydrogel flow barrier preparation

To prepare the hydrogel flow barrier serving as a flow resistor within the device, 1 ml of 4% UltraPure™ agarose hydrogel was pipetted into a 10 ml syringe sealed with a 3D-printed tip and allowed to solidify for 10 min at room temperature (RT). The seal tip was then removed, and the syringe was connected to the other 3D-printed components to assemble the pumpless flow device. The needle used at the top of the chip for air circulation was 25 G, while the needle at the outlet of the culture chamber was 27 G. 10 ml syringes (BD Microlance, USA) were used for all parts of the chip.

### Flow characterization and computational modeling

To evaluate the flow rate, assembled chips were filled via their reservoir syringes with 5 ml of DW stained with blue food coloring and operated without reservoir replenishment. Flow rates were quantified experimentally by monitoring reservoir volume changes over a 7-day period, using the graduation markings on the syringes to determine volumetric changes over time.

Computational fluid dynamics (CFD) simulations were performed using SolidWorks Flow Simulation (Dassault Systèmes, Waltham, MA, USA; version 2025) to estimate hydrostatic pressure distributions within the device. Simulations assumed laminar, incompressible flow of a Newtonian fluid with properties corresponding to standard cell culture medium. Atmospheric pressure boundary conditions were applied at the inlet and outlet. Velocity magnitude distributions were simulated using Autodesk CFD (Autodesk, Inc.; version 2024) under the experimentally measured inlet flow rate. The downstream 4% (w/v) agarose element was included as a hydraulic flow resistor to stabilize perfusion; however, velocity and pressure values reported for the culture chamber correspond to the fluid domain upstream of the hydrogel.

### Assessment of metabolic function and spheroid size under flow and static conditions

For metabolic function assays and size imaging, Ovcar-8 cells were seeded in UltraPure agarose molds at a density of 5000 cells per microwell within 3D-printed templates. After 24 h, spheroids had formed and were cultured under two conditions: (1) static control, in which the 3D-printed templates were placed in standard 24-well culture plates, and (2) flow conditions, in which the 3D-printed templates containing spheroids were placed in the fully assembled flow device. After 3 days of culture, spheroid metabolic activity and size were evaluated.

For metabolic function assessment, PrestoBlue reagent (Sigma Aldrich, MO, USA) was applied according to the manufacturer's instructions. Fluorescence was measured at excitation 530/25 nm and emission 590/20 nm using a Wallac 1420 VICTOR plate reader. Spheroids were imaged after 3 days using a Nikon Eclipse Ti microscope, and the spheroid area was quantified using the NIS Elements software.

### Statistics and reproducibility

Statistical analyses were performed using GraphPad Prism 9 (GraphPad Software, San Diego, CA, USA). All experiments were conducted with at least three independent replicates. Comparisons between two groups were analyzed using an unpaired two-tailed Student's *t*-test. Comparisons among three or more groups were performed using one-way analysis of variance (ANOVA) followed by Tukey's multiple comparison *post hoc* test. Differences were considered statistically significant at p < 0.05.

## SUPPLEMENTARY MATERIAL

See the supplementary material for additional invasion assay results and further flow characterization of the pump-free device.

## Data Availability

The data that support the findings of this study are available from the corresponding author upon reasonable request.
